# Mining the pre-diagnostic antibody repertoire of TgMMTV-neu mice to identify autoantibodies useful for the early detection of human breast cancer

**DOI:** 10.1186/2051-1426-1-S1-P59

**Published:** 2013-11-07

**Authors:** Sasha E Stanton, Jianning Mao, John Ladd, Lauren Rastedder, Ekram Gad, Samir Hanash, Mary L Disis

**Affiliations:** 1 Oncology, University of Washington, Seattle, WA, USA; 2 Fred Hutchinson Cancer Research Center, Seattle, WA, USA; 3 MD Anderson Cancer Center, Houston, TX, USA

## 

Breast cancer has been shown to be immunogenic and can stimulate an antibody immune response. Because of this immunogenicity, identification of early breast tumor antigens could allow identification of autoantibodies for the early detection of breast cancer. In this study, the TgMMTV-neu genetically engineered mouse model of breast cancer was used to discover pre-invasive tumor specific antigens. The mammary tumors that develop in these mice are genotypically similar to human luminal breast cancer and the mammary tumor development in these mice can be evaluated longitudinally, allowing for collection of sera prior to tumor development and identification of tumor specific antibodies representing the earliest alterations in the malignant transformation of normal mammary epithelium. We identified 6 antigens that were present in mice prior to the development of mammary tumors (Pdhx, Otud6b, Stk39, Zpf238, Lgals8, and Vps35). These proteins were associated with inflammation, autoimmunity, and cellular homeostasis. In mouse validation cohorts, detecting IgM and IgG antibody responses against a panel of three “pre-diagnostic” tumor antigens discriminated pre-diagnostic sera from non-transgenic control sera with an AUC of 0.924 (95% CI 0.81-1.0, p<0.001). We next evaluated samples obtained from the Women’s Health Initiative Study and demonstrated women with autoantibodies to the human homologues of these proteins. The Women’s Health Initiative study is an observational study following 161,808 healthy postmenopausal women to address issues of cardiovascular disease, cancer, and osteoporosis. Samples represented 188 sera, 94 from women who would eventually develop breast cancer and 94 matched controls. IgM and IgG autoantibodies to the “pre-diagnostic” antigen panel could discriminate the samples of women who eventually developed breast cancer from matched controls. The discriminatory potential of the pre-diagnostic autoantibodies was enhanced if samples were collected more than 5 months prior to diagnosis (AUC 0.68; CI 0.565-0.787, p=0.003). These data suggest that the same pre-invasive breast tumor proteins are found in mice and women and can predict individuals who will subsequently develop breast cancer. Future studies will further validate this panel of early tumor antigens as early breast biomarkers in high risk individuals.

